# Real-Time Personal Protective Equipment (PPE) Compliance and Clinical Tool Monitoring Using Generative AI: A Novel Approach for Adaptive and Automated Healthcare Surveillance

**DOI:** 10.7759/cureus.95182

**Published:** 2025-10-22

**Authors:** Manit Gupta, Rajaram Gairaboni, Andrei Lyle Bautista, Katherine Vo Brown, Bhavit Gupta, Austin Bautista, Alexander Bautista, Lady Christine Ong Sio, Shuchita Garg

**Affiliations:** 1 Anesthesiology, duPont Manual High School, Louisville, USA; 2 Anesthesiology, University of Louisville, Louisville, USA; 3 Anesthesiology, University of Louisville School of Medicine, Louisville, USA; 4 Anesthesiology, Meyzeek Middle School, Louisville, USA; 5 Anesthesiology, University of Louisville Hospital, Louisville, USA; 6 Department of Anesthesiology, Pain Medicine (Chronic Pain Management), University of Cincinnati Medical Center (UCMC) UC Health West Chester Hospital, Cincinnati, USA

**Keywords:** automation system, generative ai, hospital acquired infections, infection prevention and control, patient safety clinical compliance, personal protective equipment (ppe), technology in healthcare

## Abstract

Background: Hospital-acquired infections (HAIs) remain a critical patient safety concern, affecting one in 31 hospitalized patients daily. Non-compliance with personal protective equipment (PPE) protocols is a preventable driver. Current monitoring methods, such as manual audits and closed-circuit television (CCTV), are limited by delays, inconsistency, and reactivity. Traditional artificial intelligence (AI) systems are rigid and require retraining when protocols change.

Objective: To construct and evaluate a generative AI-driven compliance monitoring system, built with Google Gemini (Mountain View, CA, USA) on Raspberry Pi (Cambridge, UK) hardware that translates hospital rulebooks or free-text prompts into real-time enforcement logic without retraining.

Methods: The system integrated Gemini, OpenCV (Dover, DE, USA) and Streamlit (San Francisco, CA, USA) to convert natural language rules into executable logic. Performance was tested in 168 mannequin-based trials under varied conditions (skin tones, orientations, and object presence). Outcomes were compared with reference labels using accuracy, recall, specificity, F1 score, and Cohen’s Kappa.

Results: The system achieved 95.8% accuracy, 91.0% recall, 100% specificity, F1 = 0.95, and Cohen’s Kappa = 0.92. Performance was consistent across mannequin skin tones and between rulebook-derived and free-text prompts, with no false positives recorded.

Conclusion: This generative AI compliance system demonstrated strong accuracy, adaptability, and cost efficiency. Integration into hospital workflows could enable proactive real-time monitoring of evolving safety protocols, improving compliance and reducing costs relative to current methods

## Introduction

Healthcare-associated infections (HAIs) significantly increase patient morbidity, mortality, and healthcare costs [1,2]. Adherence to hand hygiene (HH) and personal protective equipment (PPE) is central to infection prevention [2-4]. However, compliance remains inconsistent, with lapses driving nosocomial infection [3,5,6].

Traditional compliance monitoring has critical limitations. Direct observation, while considered a gold standard, is resource-intensive, prone to bias, and subject to the Hawthorne effect [7-10]. Closed-circuit television (CCTV) provides objective data but is primarily retrospective and raises privacy concerns [10]. Traditional artificial intelligence (AI) surveillance relies on rigid, predefined algorithms that require retraining when guidelines change [1,11]. Collectively, these issues underscore the need for flexible, adaptive, and scalable monitoring systems.

AI has shown promise across infection prevention and safety applications. AI has improved HH compliance [9], enhanced fall detection [11-13], and supported environmental safety with disinfection robots [14]. These applications illustrate AI’s ability to deliver proactive and reliable monitoring.

Unlike prior AI-based PPE monitoring approaches, this system uses generative AI to translate unstructured compliance rules into executable monitoring logic without retraining, enabling rapid adaptation to changing protocols. Generative AI models are designed to learn patterns from large datasets and create new outputs such as text, images, or structured logic based on learned representations, allowing flexible adaptation across contexts. 

We hypothesize that a generative AI-driven compliance monitoring system can detect PPE adherence with ≥90% accuracy across varying conditions, offering a measurable improvement over conventional manual audits or static AI approaches. This study evaluates whether a generative AI system can reliably detect PPE compliance in simulated clinical scenarios, quantifying sensitivity, specificity, and inter-rater agreement.

## Materials and methods

System design

**Figure 1:**
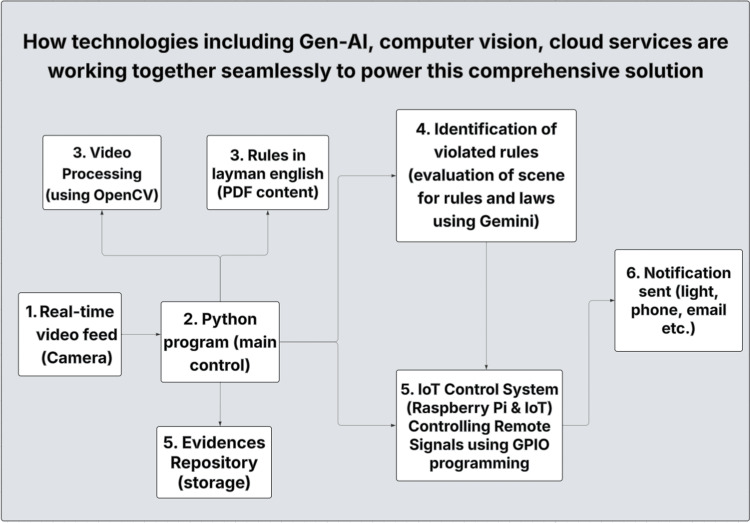
End-to-end surveillance workflow: real-time video is processed with OpenCV, analyzed by Gemini AI against user-defined rules, and triggers alerts with evidence storage. PDF: portable document format, IoT: Internet of Things, GPIO: general-purpose input/output

The system is a generative AI-driven surveillance platform built on a standard camera and Raspberry Pi 3B+ (Cambridge, UK). A real-time video feed is processed by both a main Python (Fredericksburg, VA, USA) program and OpenCV (Dover, DE, USA) for object detection. User-defined compliance rules, entered through a Streamlit (San Francisco, CA, USA) interface are sent to Google Gemini (gemini-2.0-flash-001; Mountain View, CA, USA) which interprets the natural language rules and analyzes the scene. When violations are detected, Raspberry Pi functions as an Internet of Things (IoT) controller, triggering real-time notifications via legacy devices (lights, alarms) connected using general-purpose input/output (GPIO) programming, and storing evidence (annotated images and video) for later investigation. This architecture enables low-cost deployment without new infrastructure (Figure 1).

Development and testing procedures

**Figure 2:**
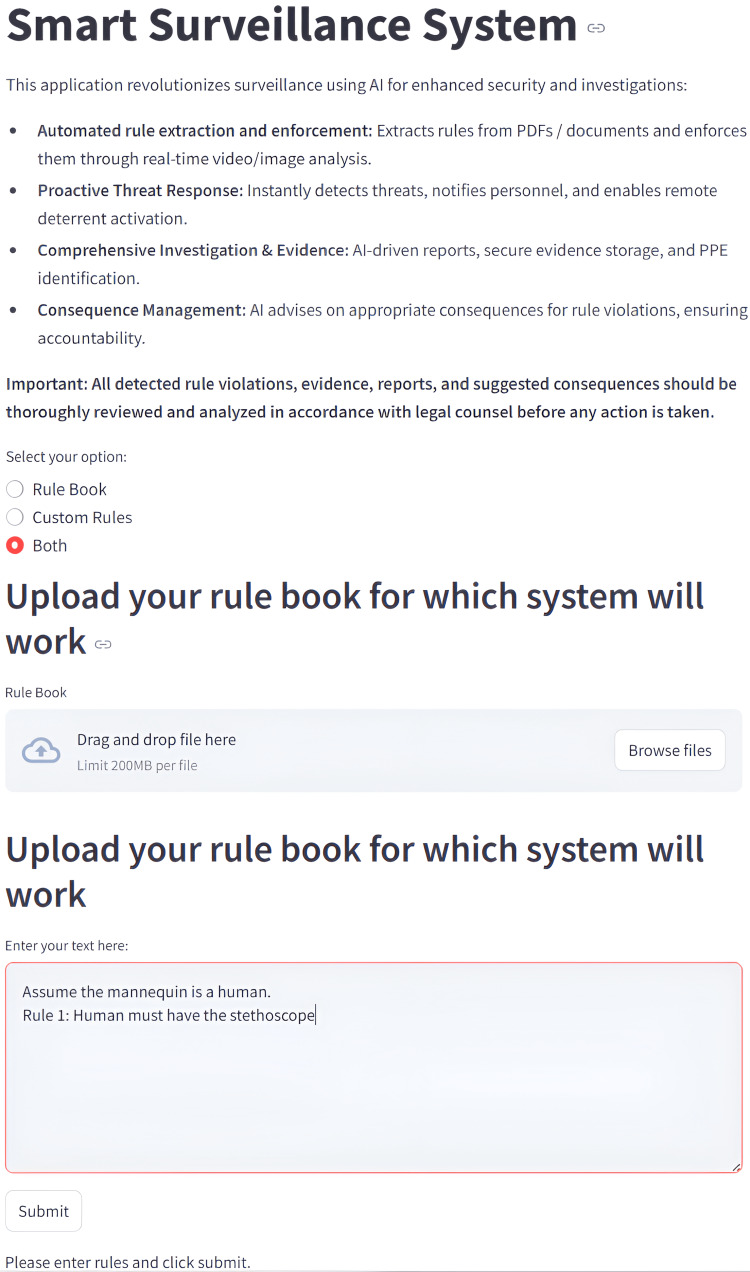
This image shows the web interface for the Smart Surveillance System, which allows users to define custom monitoring rules. The user can select to upload a portable document format (PDF) rulebook, enter custom rules directly, or both. The screenshot shows the "Both" option selected, with a sample rule entered that instructs the system to assume a mannequin is a human and requires it to be holding a stethoscope along with an option given to upload a rulebook.

A Streamlit interface enabled rule entry via free text or uploaded documents (Figure 2). Gemini translated these inputs into executable logic for OpenCV object recognition, with evidence of violations (annotated frames and text) automatically stored. Notifications were delivered through a Raspberry Pi, which activated legacy devices (lights, alarms) as intelligent outputs. Testing was conducted at three levels: functional (rule creation and detection), integration (component interaction), and notification (delivery speed). Independent variables included mannequin conditions, object type, and prompt type; dependent variables were detection accuracy and notification success. Each object was tested with two variations of the same prompt to ensure consistency across natural language variations. 

Experimental setup and evaluation

**Figure 3:**
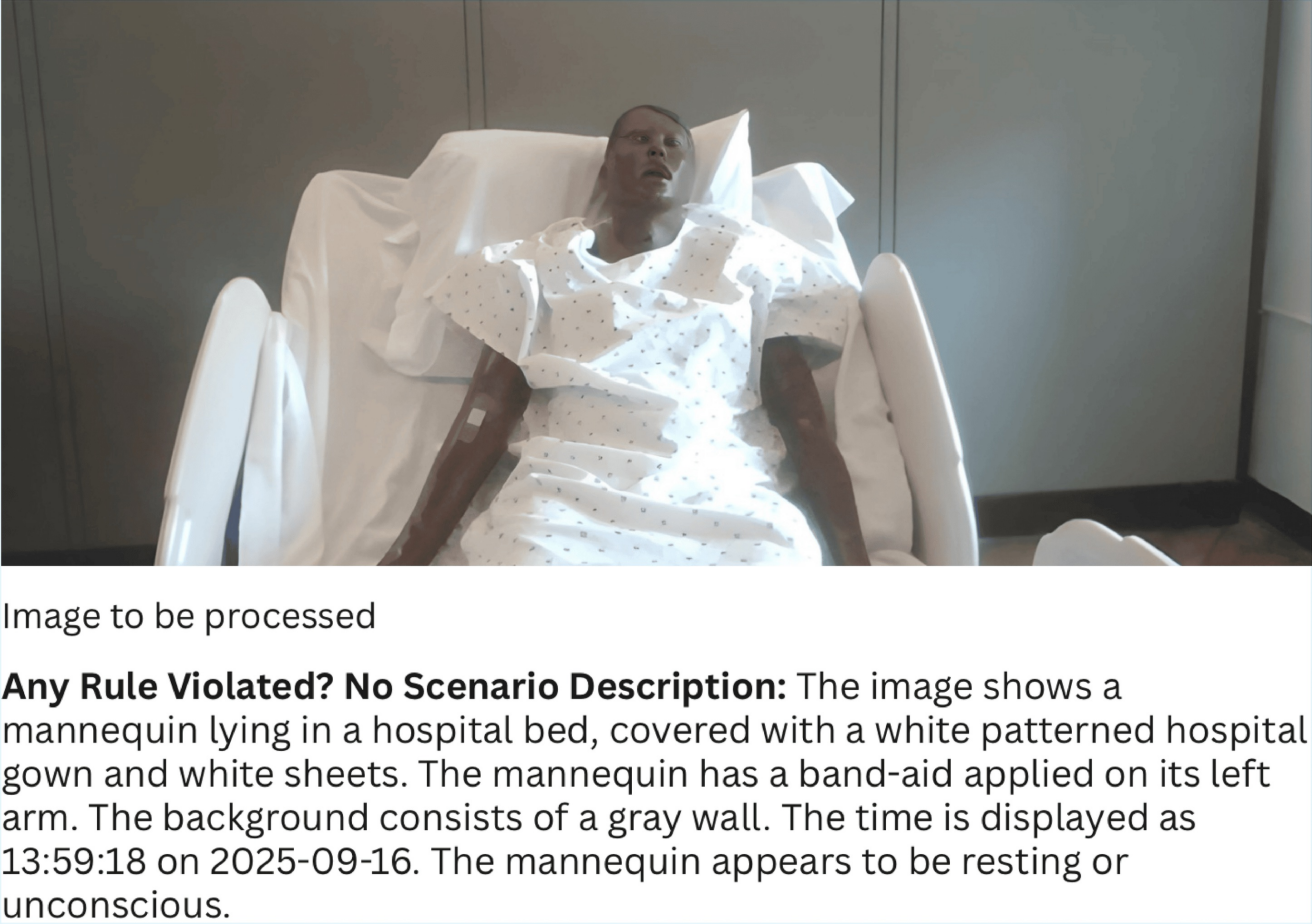
System output showing a mannequin in a hospital bed with AI analysis below, including rule evaluation, scene description, object identification, and required items. The rule given for this trial was “Assume the Mannequin is a real human. Detect if band-aid is not applied on the arm”, and the system correctly identifies it.

Trials (n=168) used mannequins of two skin tones (Black/White) and orientations (Front/Left). Objects included PPE and medical tools (e.g., gloves, gowns, stethoscopes), with both single- and multi-object scenarios (Figure 3). Two input modes were assessed: free-text prompts and Occupational Safety and Health Administration (OSHA) rulebook-derived prompts from uploaded documents. For each object-condition pair, six trials were run (three with the object present, three absent), with ground truth defined as a binary Yes/No label. Performance was evaluated using accuracy, sensitivity, specificity, F1 score, and Cohen’s Kappa, with 95% confidence intervals calculated via Wilson and bootstrap methods.

This dynamic approach allows hospitals or administrators to upload rulebooks and custom rules in various formats, which are automatically transformed into monitoring logic without requiring model retraining. This provides a highly adaptable system that can quickly accommodate new compliance criteria. The system was designed around four key criteria: real-time detection with a target accuracy of over 90%, immediate notifications, seamless integration with existing CCTV infrastructure, and a user-friendly interface. A hybrid software stack was implemented, combining Google Gemini for semantic interpretation, OpenCV for image and video stream processing, Streamlit for the user interface, and Python-based Application Programming Interface (API) for hardware and cloud integration.

## Results

Overall performance

The system demonstrated robust detection capabilities across all 168 mannequin-based trials. Overall accuracy reached 95.8% (95% CI: 91.7-98.0%), with sensitivity of 91.0% (95% CI: 82.6-95.6%) and perfect specificity of 100% (95% CI: 95.9-100%), indicating that the model reliably detected true violations while avoiding false positives. Precision was 100% (95% CI: 94.9-100%), and the F1 score, reflecting the balance between precision and recall, was 0.953 (bootstrap 95% CI: 0.911-0.986). Agreement with the reference standard was excellent, with a Cohen’s Kappa of 0.916 (Table 1). These results indicate a conservative yet highly reliable detection profile, prioritizing accuracy and precision while maintaining high recall, which is critical for minimizing unnecessary alerts and alarm fatigue in healthcare settings.

**Table 1:** Overall Detection Performance Metrics with Confidence Intervals, Cohen’s Kappa, and Matthews Correlation Coefficient This table shows the system’s overall performance across all detection tasks, with metrics calculated directly from the confusion matrix (True Positive, True Negative, False Positive, False Negative); therefore, no p-values were required, as no group comparisons were performed. Results are presented as descriptive proportions with 95% confidence intervals (Wilson’s method for proportions, bootstrap for F1 Score). The system achieved 95.8% accuracy and an F1 score of 0.953, with specificity and precision both equal to 1, indicating zero false positives. Cohen’s Kappa (0.916) and Matthews Correlation Coefficient (0.919) confirm strong agreement beyond chance, while minimal false negatives highlight high sensitivity and reliability of detection.

Measure	Value	Formula
Sensitivity	0.9103	True Positive Rate = True Positive / (True Positive + False Negative)
Specificity	1	Specificity = True Negative / (False Positive + True Negative)
Positive Predictive Value (Precision)	1	Positive Predictive Value = True Positive / (True Positive + False Positive)
Negative Predictive Value	0.9278	Negative Predictive Value = True Negative / (True Negative + False Negative)
False Positive Rate	0	False Positive Rate = False Positive / (False Positive + True Negative)
False Discovery Rate	0	False Discovery Rate = False Positive / (False Positive + True Positive)
False Negative Rate	0.0897	False Negative Rate = False Negative / (False Negative + True Positive)
Accuracy	0.9583	Accuracy = (True Positive + True Negative) / (True Positive + True Negative + False Positive + False Negative)
F1 Score	0.953	F1 = 2*True Positive / (2*True Positive + False Positive + False Negative)
Matthews Correlation Coefficient	0.919	Matthews Correlation Coefficient = (True Positive * True Negative – False Positive * False Negative) / (sqrt((True Positive + False Positive) * (True Positive + False Negative) * (True Negative + False Positive) * (True Negative + False Negative)))

Subgroup analyses

Single-object trials consistently exhibited higher sensitivity and F1 scores than multi-object combinations, though overall performance remained strong (Table 2). The AI system achieved perfect accuracy, sensitivity, specificity, and F1 scores (1.0) for most PPE and patient-safety rules-including stethoscopes, gloves, gowns, masks, blood pressure cuffs, oximeters, and bed placement were also included to evaluate the system’s versatility in detecting both PPE adherence and proper handling of clinical equipment, with no false positives observed, indicating alerts were mostly correct. Performance was lower for nuanced tasks such as band-aid placement or combined syringe and band-aid detection, with accuracy 0.5-0.83 and sensitivity 0-0.67, though specificity and precision remained high across all prompts. In rulebook trials, the numerous rules caused the system to flag violations for all missing items, making it impossible to satisfy every rule simultaneously (Table 2). Rulebook-only trials tested the mannequin without PPE, while rulebook + item trials assessed one item at a time. Consequently, F1, precision, and sensitivity are not reported for rulebook-only trials, as no scenario satisfied all rules.

**Table 2:** Detection Performance of Medical Object by Using Confusion Matrix Metrics This table evaluates system performance across various medical objects and combinations using descriptive metrics calculated from the confusion matrix (True Positive, True Negative, False Positive, False Negative). Accuracy, sensitivity (recall), specificity, precision, and F1 score were computed directly from these counts. No hypothesis tests or p-values were performed, as the table reports descriptive proportions for each object rather than comparisons between groups; confidence intervals were not included, as the purpose is to summarize overall detection performance. Many objects (e.g., gloves, gown, mask) achieved perfect detection (100% accuracy and F1 score), whereas smaller or combined items (e.g., bandaid, bandaid+syringe) showed reduced sensitivity (0.33–0.50), highlighting detection challenges.

Label	Total	True Positive	False Negative	True Negative	False Positive	Accuracy	Sensitivity (Recall)	Specificity	Precision	F1 Score	Balanced Accuracy
Object: Blood Pressure Cuff	6	3	0	3	0	1	1	1	1	1	1
Object: Blood Pressure Cuff	6	3	0	3	0	1	1	1	1	1	1
Object: Bandaid	12	3	3	6	0	0.75	0.5	1	1	0.6666666667	0.75
Object: Bandaid + Syringe	12	2	4	6	0	0.6666666667	0.3333333333	1	1	0.5	0.6666666667
Object: Gloves	12	6	0	6	0	1	1	1	1	1	1
Object: Gown	12	6	0	6	0	1	1	1	1	1	1
Object: Gown + Socks	6	3	0	3	0	1	1	1	1	1	1
Object: Gown+socks	6	3	0	3	0	1	1	1	1	1	1
Object: Mask	12	6	0	6	0	1	1	1	1	1	1
Object: On floor	12	6	0	6	0	1	1	1	1	1	1
Object: Oximeter	6	3	0	3	0	1	1	1	1	1	1
Object: Oximeter	6	3	0	3	0	1	1	1	1	1	1
Object: Oximeter + Gown + Blood Pressure Cuff	12	6	0	6	0	1	1	1	1	1	1
Object: Rulebook	12	0	0	12	0	1		1			1
Object: Rulebook + rule glove	12	6	0	6	0	1	1	1	1	1	1
Object: Rulebook + rule mask	12	6	0	6	0	1	1	1	1	1	1
Object: Stehoscope	6	3	0	3	0	1	1	1	1	1	1
Object: Stethoscope	6	3	0	3	0	1	1	1	1	1	1

System performance across skin tones was robust: Black and White mannequins showed overlapping confidence intervals for sensitivity, specificity, and F1 scores, indicating negligible bias and equitable detection (Table 3).

**Table 3:** Detection Performance by Mannequin Skin Tone Using Confusion Matrix Metrics with Confidence Intervals and Cohen’s Kappa This table reports system performance across two mannequin skin tones (Black and White) using descriptive metrics derived from the confusion matrix (True Positive, True Negative, False Positive, False Negative). Accuracy, sensitivity (recall), specificity, precision, F1 score, and balanced accuracy were calculated directly from these counts, with 95% confidence intervals estimated using Wilson’s method for proportions and bootstrap resampling for the F1 score. Cohen’s Kappa was also computed to assess agreement beyond chance, indicating high reliability across both skin tones. No p-values or hypothesis tests were applied in this table, as the purpose is to summarize performance descriptively rather than to compare groups statistically. Performance was strong overall, with Black mannequins achieving 0.976 accuracy and 0.974 F1 score, and White mannequins achieving 0.940 accuracy and 0.932 F1 score, demonstrating minimal bias in detection.

Label	Total	True Positive	False Negative	True Negative	False Positive	Accuracy	Sensitivity (Recall)	Specificity	Precision (PPV)	F1 Score	Balanced Accuracy
Skin: Black	84	37	2	45	0	0.9761904762	0.9487179487	1	1	0.9736842105	0.9743589744
Skin: White	84	34	5	45	0	0.9404761905	0.8717948718	1	1	0.9315068493	0.9358974359

Similarly, performance across camera positions was consistently high, with accuracy >95% and perfect specificity. Slightly lower sensitivity in side-angle trials suggests minor variation, but confidence intervals overlapped, confirming robustness across viewing perspectives (Table 4). 

**Table 4:** Detection Performance by Camera Position with Confidence Interval and Cohen’s Kappa This table summarizes system performance across two camera positions (front and left) using descriptive classification metrics calculated from the confusion matrix (True Positive, True Negative, False Positive, False Negative). Accuracy, sensitivity (recall), specificity, precision, F1 score, and balanced accuracy were computed directly from these counts. Cohen’s Kappa was also calculated to quantify agreement beyond chance, and 95% confidence intervals for proportions were estimated using Wilson’s method. No hypothesis tests or p-values were performed, as the table reports descriptive performance metrics rather than comparisons between groups, so statistical significance was not required. Performance remained high for both positions, with slightly lower sensitivity for the left-angle trials (0.885), but specificity and precision remained perfect (1.0), demonstrating reliable detection across camera angles.

Label	Total	True Positive	False Negative	True Negative	False Positive	Accuracy	Sensitivity (Recall)	Specificity	Precision (PPV)	F1 Score	Balanced Accuracy
Position: Front	84	48	4	32	0	0.9523809524	0.9230769231	1	1	0.96	0.9615384615
Position: Left	84	23	3	58	0	0.9642857143	0.8846153846	1	1	0.9387755102	0.9423076923

Overall, false negatives were limited, and no false positives were observed, reflecting a conservative detection strategy. Multi-object scenarios and certain positional angles were the most challenging, yet overall performance remained strong. Rulebook integration proved advantageous for consistent compliance enforcement, supporting the system’s practical utility in real-world hospital settings.

## Discussion

This study demonstrates that a generative AI-based compliance monitoring system can reliably detect PPE adherence and other healthcare safety behaviors, such as the correct handling and placement of clinical tools including stethoscopes, blood pressure cuffs, and oximeters, in real time, achieving high accuracy and excellent agreement with reference standards (Cohen’s Kappa = 0.92). Detection performance was consistent across mannequin skin tones, camera orientations, and prompt types, highlighting fairness and potential generalizability. Importantly, no false positives were observed, minimizing unnecessary alerts that could contribute to alarm fatigue, while sensitivity remained high at 91%, indicating effective detection of true violations.

Compared with manual audits, CCTV monitoring, and conventional AI systems, the system offers significant advantages: proactive monitoring, dynamic interpretation of evolving compliance rules without retraining, and integration with existing infrastructure via low-cost Raspberry Pi hardware [1-3,10]. Clinically, this enables real-time enforcement of infection prevention measures such as PPE adherence, hand hygiene, operating room sterile protocols, and safe handling of medical equipment, while reducing hospital-acquired infections, labor-intensive observation, associated costs, and the workload for infection prevention and control nurses.

**Figure 4:**
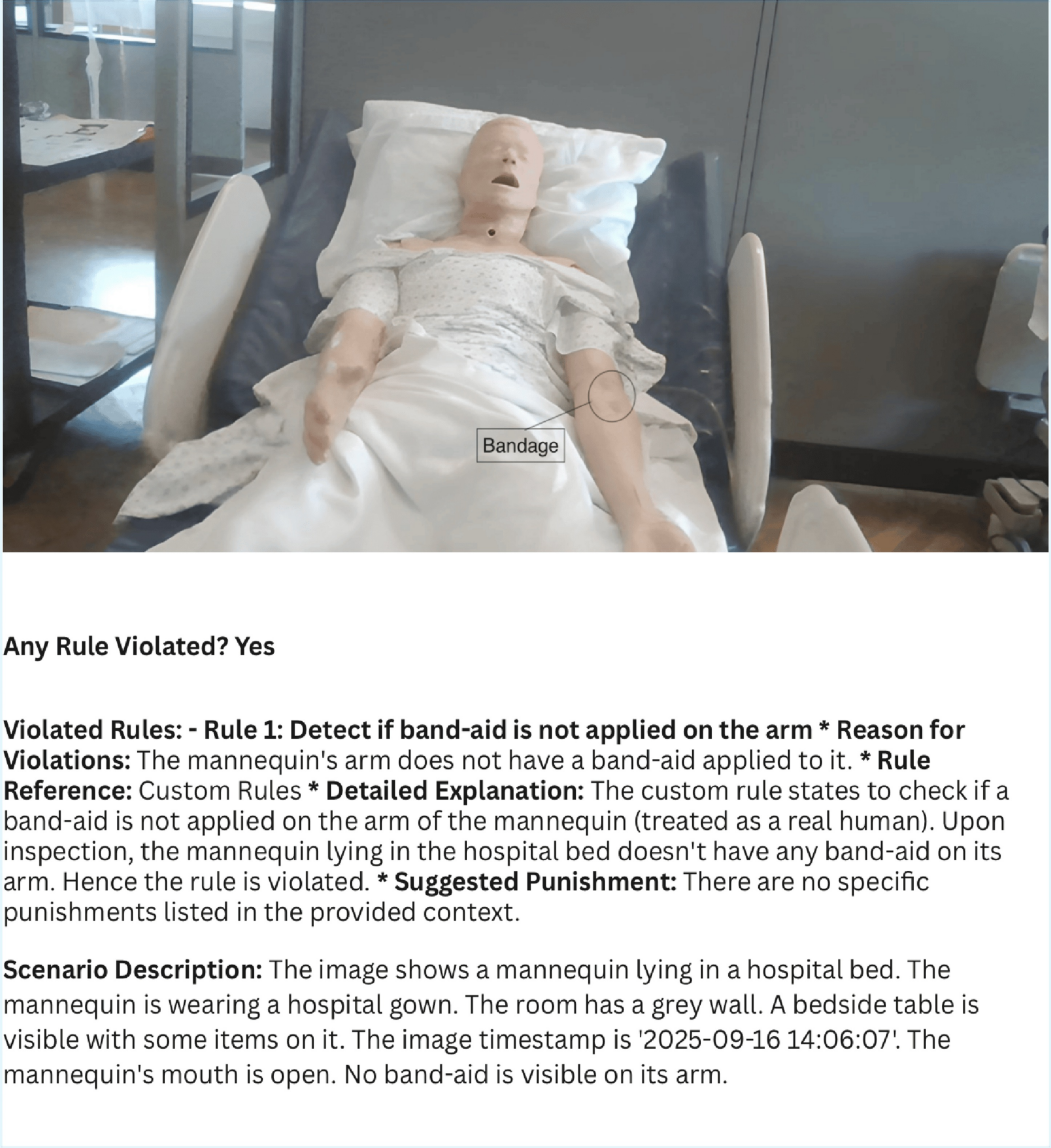
The image shows a mannequin in a hospital bed, with a bandage, serving as the input for the project. The analysis correctly notes the mannequin's appearance but provides an incorrect result, flagging a rule violation for a "missing" band-aid, even though one is present.

Limitations include mannequin-based testing under controlled conditions, sensitivity below 100%, and challenges in multi-object detection, camera distance, or objects blending with backgrounds (Figure 4). Additionally, real-time monitoring systems raise important ethical concerns related to privacy and potential HIPAA violations. Future work should expand datasets, conduct live hospital trials with cost-benefit analyses, integrate with electronic health records and alert systems, and optimize detection under more complex environmental conditions. 

Overall, this generative AI system combines adaptability, fairness, and cost-efficiency, offering a robust solution for real-time compliance monitoring. Its consistent performance across object combinations, skin tones, and camera angles demonstrates strong generalizability, highlighting its potential for dynamic hospital environments. By enabling proactive detection of PPE and safety protocol adherence, the system can enhance patient and staff safety, reduce reliance on manual audits, and support scalable, automated infection prevention.

## Conclusions

This generative AI-driven surveillance system demonstrated high accuracy, reliability, and fairness in monitoring PPE and healthcare compliance behaviors across different mannequin skin tones, orientations, and prompt types. Compared with manual audits, CCTV monitoring, and conventional AI models, it offers a proactive, flexible, and cost-effective approach that can dynamically incorporate evolving protocols without retraining. While testing was limited to mannequin trials under controlled conditions, and sensitivity below 100% indicates a small risk of missed detections, the results support further hospital-based trials and integration with clinical systems. With additional validation, this system has the potential to enhance real-time compliance monitoring, reduce infection risk, and improve patient and staff safety.
